# Strategies for the Preparation of Chitosan Derivatives for Antimicrobial, Drug Delivery, and Agricultural Applications: A Review

**DOI:** 10.3390/molecules28227659

**Published:** 2023-11-18

**Authors:** Rajeev Shrestha, Anusree Thenissery, Rahul Khupse, Gireesh Rajashekara

**Affiliations:** 1 Center for Food Animal Health, Department of Animal Sciences, The Ohio State University, Wooster, OH 44691, USA; thenissery.1@buckeyemail.osu.edu; 2 College of Pharmacy, University of Findlay, Findlay, OH 45840, USA; khupse@gmail.com

**Keywords:** chitosan (CS) derivatives, antimicrobial activity, drug delivery, infectious diseases, plant agriculture

## Abstract

Chitosan has received much attention for its role in designing and developing novel derivatives as well as its applications across a broad spectrum of biological and physiological activities, owing to its desirable characteristics such as being biodegradable, being a biopolymer, and its overall eco-friendliness. The main objective of this review is to explore the recent chemical modifications of chitosan that have been achieved through various synthetic methods. These chitosan derivatives are categorized based on their synthetic pathways or the presence of common functional groups, which include alkylated, acylated, Schiff base, quaternary ammonia, guanidine, and heterocyclic rings. We have also described the recent applications of chitosan and its derivatives, along with nanomaterials, their mechanisms, and prospective challenges, especially in areas such as antimicrobial activities, targeted drug delivery for various diseases, and plant agricultural domains. The accumulation of these recent findings has the potential to offer insight not only into innovative approaches for the preparation of chitosan derivatives but also into their diverse applications. These insights may spark novel ideas for drug development or drug carriers, particularly in the antimicrobial, medicinal, and plant agricultural fields.

## 1. Introduction

**Scheme 1:**
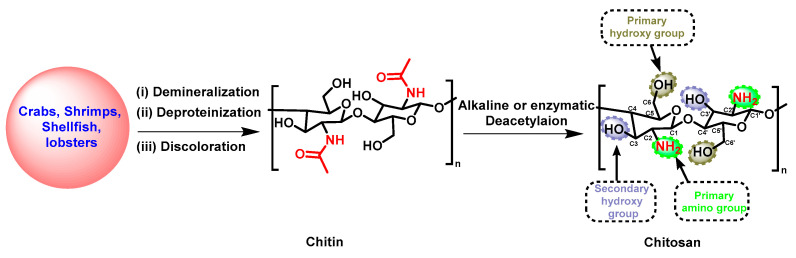
Synthetic strategy for the preparation of CS.

Over the last few decades, natural polymers, including biopolymers, polyesters, and proteins, have displayed diverse applications in the fields of agriculture, pharmaceuticals, food safety, and the cosmetic industry due to their biodegradability, eco-friendliness, compatibility, and cost-effectiveness [1,2,3]. As a result, natural polymers have gained significantly increased interest in creating novel drugs, owing to their distinct and intricate chemical composition, which is responsible for their wide and diverse range of biological effects [4,5]. Among these, chitosan (CS) stands out as a widely utilized polysaccharide that has been extensively studied across various scientific disciplines [6,7,8]. CS is composed of linear polysaccharides with varying amounts of (β1 → 4) linked residues of *N*-acetyl-2 amino-2-deoxy-D-glucose (glucosamine, GlcN) and 2-amino-2-deoxy-D-glucose (*N*-acetyl-glucosamine, GlcNAc) residues distributed inside the polymer. It is derived from chitin, polyβ-[1,4]-*N*-acetyl-d-glucosamine) (or poly(*N*-acetyl-d-glucosamine) via deacetylation in the presence of sodium hydroxide or enzymes (Scheme 1) [9,10,11]. Chitin is found in crustaceans, including crabs, shrimp, shellfish, and lobsters, and its extraction primarily involves demineralization, deproteinization, and discoloration [12,13]. Its hydrophilic and reactive properties are limited by the presence of acetyl groups (CH_3_CO^−^) attached to the amino groups of GlcNAc. These acetyl groups function not only as barriers for water molecules, but also as protective shields, which reduce the availability of the amino groups for reactions [14]. For the biological and physical activity of CS, the degree of deacetylation (DD) is the pivotal factor, which represents the proportion of β-1,4-D-glucosamine repeating units in the polysaccharides during the deacetylation process originating from chitin [9,10,11,14]. A higher DD denotes an increased presence of free amino groups, which can form ammonium (NH_4_^+^) groups within a pH range of 5–6, resulting in increased water solubility and the enhancement of the biological impact of CS [15]. Furthermore, a lower molecular weight (MW) is another factor that decreases with a higher DD, which impacts the bioactivity of CS [15]. CS with a lower MW has demonstrated antibacterial, antioxidant, and anti-tumor properties due to its increased water solubility, ease of permeability to cells, and binding efficacy to DNA or RNA [16,17].

CS is a cationic biopolymer agent that can interact with negatively charged substances such as lipids, fats, ions, cholesterol, proteins, and other organic and inorganic molecules [18]. CS was first identified in 1811 in mushrooms. Extensive research has revealed that CS significantly exhibits antimicrobial, antiviral, anticancer, non-toxic, water treatment, biocompatible, and biodegradable properties [19,20]. However, its solubility in acidic conditions (pH less than 6) limits certain bioactivities and applications. To address these issues and to enhance the bioactive and physiochemical effectiveness under neutral and physiological conditions, the modification of CS is a promising alternative since it has three active functional groups: C2-NH_2_, C3-OH, and C6-OH (Scheme 1) [21]. The nucleophilic substitution reactions involving the amino and hydroxyl groups, followed by multi-step reactions, have been studied, including *N*-alkylation/amination, acetylation, cross-linking, oxidation, grafting, and sulfation [22,23]. Synthetic reactions are accomplished through chemical or enzymatic catalysis, polymerization, or hydrolysis conditions to achieve the desired product [24]. The CS derivatives, which vary in terms of their degree of substitution (DS), a crucial factor in the structure–activity relationship (SAR), demonstrate unique physiochemical characteristics. Novel scaffolds with these characteristics have enhanced biocompatibility, bioactivity, biodegradability, and non-toxicity, while retaining the inherent pharmacological effects of their antibacterial, anticancer, and antiviral properties [25]. Moreover, CS derivatives have also been widely utilized in the field of nanomaterials due to their immense potential as carriers for targeted drugs, adjuvants, or vaccines, facilitating the treatment of various diseases including cancer, diabetes, Alzheimer’s, cardiovascular disorders, and inflammatory conditions [26,27]. These nanomaterials have shown superior outcomes compared to conventional drug formulations.

Herein, we explore the recent progress of CS derivatives, focusing on modifications to their functional groups conjugated to the polymer skeleton as well as substitutions in the active sites, including C2-NH_2_, C3-OH, and C6-OH. Importantly, we discuss the synthesis process of CS derivatives, categorizing them into various chemical groups based on their synthetic pathway or functional characteristics, which included alkylated, acylated, Schiff base, quaternary ammonia, guanidine, and heterocyclic CS derivatives. Moreover, this review also comprehensively covers the diverse applications of CS and its derivatives as nanomaterials in the antimicrobial, agricultural, and drug delivery fields, including the elucidation of their mechanisms of action and the factors that contribute to superior outcomes compared to conventional approaches.

## 2. Global Research Trends of Chitosan

The global research trends surrounding CS underscore its significant scientific and technological value. As projected by Precedence Research, the CS market is expected to surpass US $29 billion by 2030, reflecting its substantial importance across various sectors [28]. These trends are driven by several key factors. Notably, there is a growing demand for CS in various end-user industries, an increasing emphasis on global water treatment activities, significant advancements in healthcare and the medical sector in industrialized countries, and a heightened awareness of public health concerns, including rising obesity rates. The Asia–Pacific region has assumed a prominent position, dominating the CS market share [28]. Over the last two decades, CS has ascended to a leading role in research involving biomedicine, nanomedicine, and cell and tissue engineering [29]. The increase in research endeavors is well noted by the heightened number of patents, particularly in the biomedical sector, where its gelling properties enable viscosity and density adjustments under specific physiochemical conditions to enhance biocompatibility [30] (Table 1). Furthermore, the applications of CS underscore these global research trends. CS finds utility in diverse fields, which have extended constant research efforts in drug delivery, dentistry, ophthalmology, wound dressings (some marketed CS-based wound dressings: HemCon^®^ Bandage PRO, HemCon ChitoFlex^®^ PRO, Chitopack C, ChiGel, Tegaderm, Tegasorb, and Traumastat), cell encapsulation, bioimaging, tissue engineering, food packaging, gelling and coating, abiotic stress prevention in plants, enhanced water availability in crops, controlled-release fertilizers, dye-sensitized solar cells, wastewater and sludge treatment, and metal extraction [31,32]. This multifaceted use highlights CS’s pivotal role in shaping global research trends across healthcare, agriculture, and various industries on a worldwide scale, making it a central focus in the realm of biopolymers.

## 3. Preparation Methods of Chitosan Derivatives

Owing to the efficient biological and physiochemical properties of CS derivatives, various pathways of CS modification have been explored. The methods involve single- to multi-step reactions, including direct modification, catalytic or enzymatic reactions, chemical grafting, and cross-linking reactions under various conditions [23]. In terms of the chemical reactivity of CS, 2-NH_2_ displays more reactivity compared to the OH group. Therefore, to achieve the selective functionalization of the OH group, NH protection by using the phthaloyl group has been employed [48,49,50]. However, OH protection groups, including triphenylmethyl [51], trimethylsilyl [52], and tertiary-butyl dimethylsilyl [53,54], have also been utilized to achieve the specific functionalization of the NH group, resulting in a high DS in the products.

Although CS is soluble in acidic water through the binding process of H^+^ with the N atom of amino group, CS exhibits a high degree of crystallinity due to the intermolecular and intramolecular hydrogen bonding, leading to its near insolubility in normal water [55]. Therefore, the key benefit of CS modification is the enhancement of the water solubility of CS in high pH ranges by introducing hydrophilic groups like amino, carbonyl, carboxyl, hydroxyl, or sulfhydryl groups into the CS [55,56,57,58,59]. These functional groups are capable of not only destroying the original hydrogen bonding and crystallinity of CS but also creating new hydrogen bonds with water, thereby increasing the solubility of the product. Consequently, the modified CS is often employed to improve the solubility of the product, demonstrating the synergetic efficacy of attached functional groups for antimicrobial, antifungal, and other biological activities [23,24]. The researchers have employed CS with a wide range of MWs (ranging from 50 to 1400 kDa) and DDs (ranging from 70 to 95%) in their experiments to produce CS derivatives with various ranges of DSs of the functional group. A higher DS leads to better water solubility and higher biological potentials [29,60].

### 3.1. Alkylation, Acylation, and Schiff Base-Based Chitosan Derivatives

**Scheme 2:**
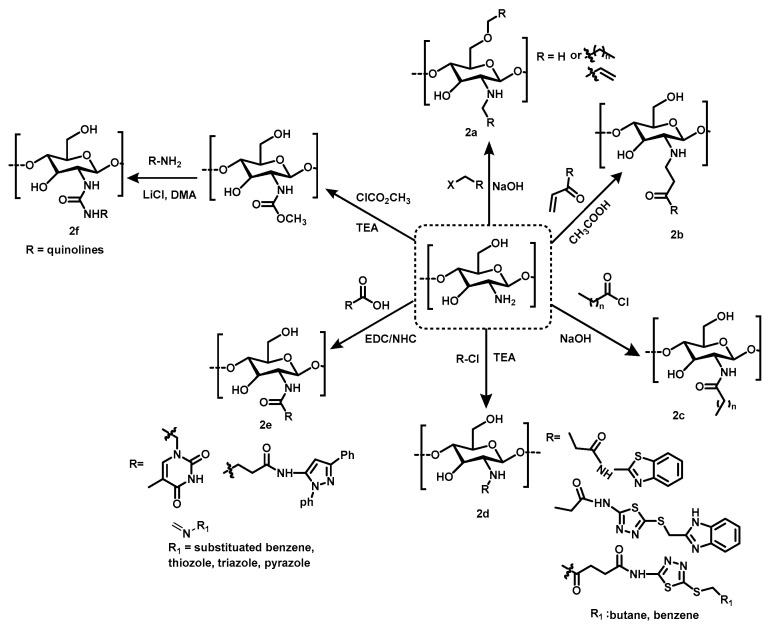
Synthesis scheme of chitosan derivatives through alkylation and acylation processes.

The alkylation of CS entails the hydrophobic modification of this material. The resulting hydrophobic traits yield specific benefits, especially the restriction of liquid interchange between the external surroundings and wound tissue. This property creates a protective barrier on the wound surface that impedes the infiltration of airborne bacteria and upholds gas exchange, ultimately leading to rapid wound healing [61]. The alkylation of CS (**2a**) is conducted by employing various alkyl or allyl halides such as bromoethane, bromohexane, bromododecane, bromohexadecane, chlorobutane, and allyl bromide in the presence of a strong base like sodium hydroxide (Scheme 2) [62,63,64]. However, the Nie group reported that the Michael addition reaction of CS with hydroxyethylacryl in acidic media led to alkylated CS (**2b**) [65]. On the other hand, CS has been acylated with various acyl chlorides in the presence of NaOH, with the initial step involving the dissolution of CS in acetic acid with water (**2c**) [66,67]. The acylation reaction has the potential to disrupt both the intramolecular and intermolecular H-bonding within the CS, leading to a reduction in its crystallinity and enhancing water solubility [68]. In recent studies, researchers have explored the reaction of CS with pre-prepared acyl chlorides or acetic acid containing diverse heterocycles like benzothiazole, thiadiazole, thymine, or pyrazoles to produce the acylated CS bearing the heterocycles (**2d**, **2e**) [69,70,71,72,73,74,75,76]. Similarly, Zhang’s group developed CS-bearing quinolinyl urea derivatives (**2f**). The reaction was accomplished by initiating a reaction between methyl chloroformate and CS, followed by a subsequent reaction with 2-aminoquinoline [77]. Specifically, the incorporation of heterocyclic groups into CS, with substitution degrees of 57.4% [75] and 47% [76], resulted in enhanced antimicrobial activities.

**Scheme 3:**
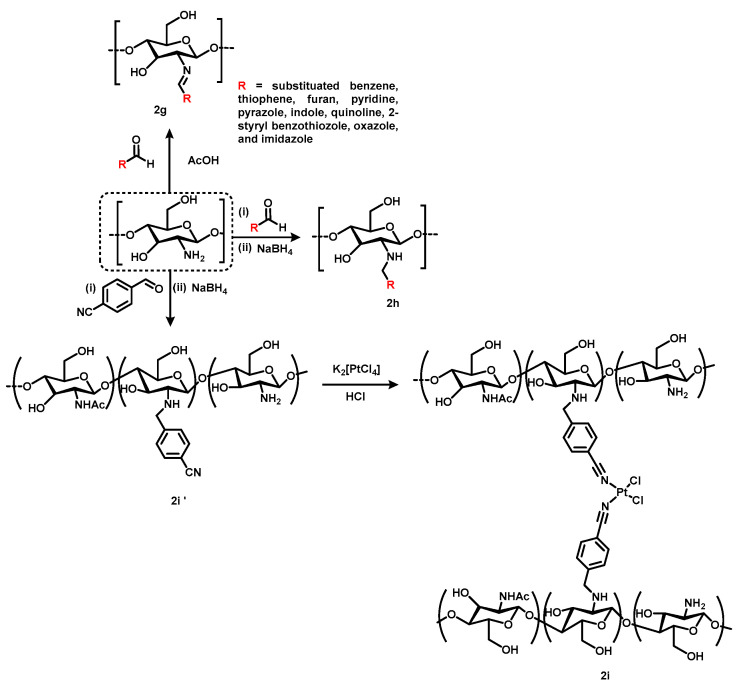
Synthesis of chitosan derivatives bearing Schiff bases.

In the course of the modification of CS derivatives by using CS (MW: 100–300 kDa, DD: 74–95%), the preparation of Schiff bases emerges as the most straightforward method. This approach includes the reaction of an amine group with benzaldehydes through an intermediate carbinolamine (Scheme 3). This method not only facilitates the introduction of new groups, such as heterocycles, but also enables subsequent alkylation after reduction [78,79]. In a plethora of studies, the attachment of heterocycles such as thiophene, furan, pyrazole, indoles, quinolone, benzothiazole, oxazole, and imidazole-bearing Schiff base CS derivatives (**2g**; DS: 1.15–56%) was extensively explored in acetic acid as a mild condition [80,81,82,83,84]. Incorporating heteroatoms into CS as Schiff base resulted in a hydrophobic characteristic; nevertheless, the presence of pyrazole and thiophene groups exhibited water solubility for CS [80,81,82,83,84]. Additionally, the reduction of Schiff bases with sodium borohydride has been widely employed as an alternative alkylation strategy, leading to the attachment of new functional groups or heterocycles (DS: 36–81%) (**2h**) [85]. Moreover, Khrustalev et al. revealed innovative CS derivatives with novel heterocyclic structures (**2i**), forming complexes with platinum metals [86]. Herein, CS of various MWs (viscosity average MW: 3.7 × 10^4^ D to 17.8 × 10^4^ D) was employed to introduce nitrile derivatives of CS (**2i’**) in the range of DS, 17% to 59%. This synthesized platinum complex was not soluble in water; however, the 1,2,4-oxadiazoline CS derivatives prepared from nitrones, a functional group consisting of *N*-oxide and imine, were soluble in water and showed a high antibacterial activity coupled with low toxicity [86].

### 3.2. Quaternary Ammonium Chitosan Derivatives

**Scheme 4:**
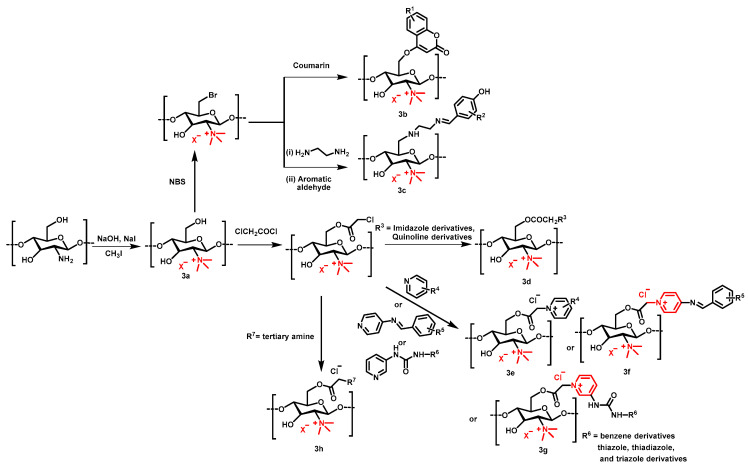
Synthesis of quaternary ammonium salt, *N*-trimethyl chitosan derivatives. The red color represents the functional group of quaternary ammonium salt.

Quaternary ammonium CS derivatives are the modified form of CS salt bearing a positively charged nitrogen atom, covalently attached with alkyl or aryl groups. The hydrophilic quaternary ammonium group enhances the water solubility of CS by introducing a quaternary ammonium salt group. The salt increases the electrostatic charge and weakens intramolecular hydrogen bonds, resulting in a high level of water solubility [87]. This quaternary ammonium salt improves not only the hydrophilicity of the final CS derivatives but also enhances its antimicrobial properties, making it valuable in diverse applications, including wound dressings, drug carriers, and other biomedical fields [88]. In addition, the polymeric quaternary ammonium CS bearing heterocyclic compounds has attracted a significant antimicrobial agent due to the synergetic effects of CS, quaternary ammonia salts, and heterocyclic compounds [89]. Commonly, the quaternary ammonium salt, *N*-trimethyl CS (TMC, **3a**), is prepared via the methylation of CS (MW: 5–200 kDa; DD: 73–97%) in the presence of sodium iodide and iodomethane under alkaline conditions (Scheme 4) [90,91,92,93,94,95,96,97,98,99]. However, there is the possibility of *O*-methylation, which can be reduced by using either an acidic or heterogeneous medium. The inclusion of heterocyclic and aromatic moieties shows promising prospects for drug development [91]. By taking advantage of this, TMC has been further subjected to derivatization, enabling the incorporation of heterocyclic and aromatic groups. In this respect, Guo et al. prepared the intermediate 6-bromo-6-deoxy-*N*-trimethyl quaternary ammonium CS through the bromination of *N*-bromobutanimide (NBS) on TMC. Subsequently, it was further reacted with coumarins with electron-donating or electron-withdrawing groups under basic conditions to yield coumarins bearing TMC (DS: 69–82%) (**3b**) [92]. In another study of their group, 6-bromo-6-deoxy-*N*-trimethyl quaternary ammonium CS was further investigated by treating it with ethylenediamine and mono- or bi-substituted polyhydroxyl benzaldehyde in two steps, leading to the formation of phenol or polyphenol-containing trimethyl quaternary ammonium CS (**3c**) [93].

In the meantime, an alternative approach has been introduced to prepare *O*-chloroacetyl quaternary ammonium CS, which has been further employed in chemical modifications involving heterocyclic compounds due to its high effectiveness in nucleophilic substitution reactions (Scheme 4). For example, compounds such as quinoline (DS: 24.34–98.7%), imidazole (DS: 12.7–65.5%), amino pyridines (DS: 72–82%), pyridines, substituted pyridines bearing Schiff bases (DS: 21.5–58.3%) or urea derivatives (DS: 21–91%), and tertiary amines (DS: 45.3–84.3%) were subjected to reactions with *O*-chloroacetyl quaternary ammonium CS, resulting in the formation of double quaternary ammonium CS salt derivatives (**3d**–**3h**) [96,97,98,99]. The advantages of double-quaternary ammonium salt include making CS more cationic and highly soluble in water.

**Scheme 5:**
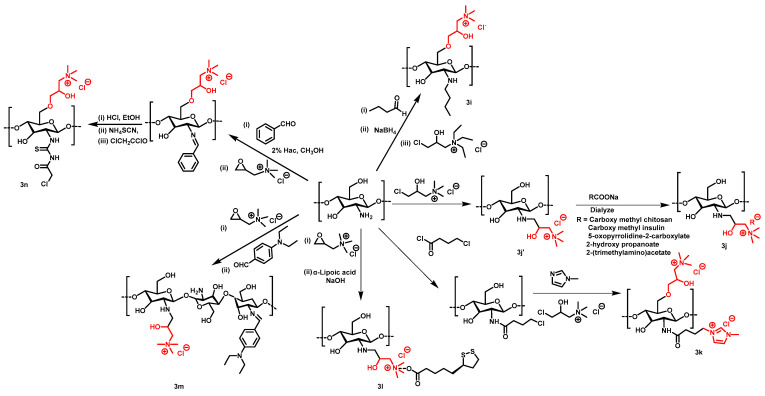
Synthesis of chitosan quaternary ammonium derivatives through a quaternary ammonium source. The red color represents the functional group of quaternary ammonium salt.

While developing the novel derivatives of quaternary ammonium CS, the water-soluble (3-Chloro-2-hydroxypropyl) trimethy/triethyl ammonium chloride (CHTAC) or glycidyl trimethyl ammonium chloride serves as an excellent quaternary ammonium source, which has been employed in nucleophilic substitution reactions, either C2-NH_2_ or C6-OH, of CS (Scheme 5) [100,101,102,103,104,105,106,107,108]. In recent years, *n*-alkyl quaternary ammonium CS (**3i**) and 2-hydroxypropyl trimethyl ammonium chloride CS (**3j’**) were synthesized through the reaction of *n*-alkyl CS or CS with a quaternary ammonium source, CHTAC. The *n*-alkyl CS was initially obtained through the alkylation of CS with *n*-butylaldehyde using the Schiff alkali method, followed by reduction using sodium borohydride [100]. The optimal synthesis condition for a higher DS (70–90%) of **3i** was optimized in various temperatures, molar ratios of chemicals, and reaction times. While the extended *n*-alkyl chain notably reduces the intermolecular hydrogen bonds in CS, it alone does not lead to complete dissolution in water. However, when combined with quaternary ammonium salt, it significantly enhances water solubility [100]. On the other hand, **3j’** was further dialyzed with carboxymethyl insulin [101] and other acetate derivatives against deionized water [102], followed by lyophilization to obtain the desired quaternary ammonium CS derivatives (**3j**; DS: 17.5–40.9% and 46–81%). In the meantime, the Zheng group also explored imidazole-based quaternary ammonium CS derivatives (DS: 36%) through multi-step nucleophilic substitution reactions [103]. The synthesis process comprised a sequential generation of intermediary compounds, *N*-chlorobutanol CS and *N*-(1-carboxybutyl-4-(3-methyl-imidazole) CS chloride, through 4-chlorobutanol chloride and *N*-methylimidazole. The ultimate product (**3k**) was obtained through the reaction between CHTAC and *N*-(1-carboxymethyl-4-(3-methyl-imidazole)) CS chloride. Likewise, hydroxypropyl trimethyl ammonium chloride CS (HACC) has demonstrated itself as an excellent water-soluble quaternary CS derivative, which is commonly prepared from the reaction of CS and glycidyl trimethyl ammonium chloride in deionized water [104,105]. Furthermore, the introduction of thioctate [106] and Schiff base [107] functionalities onto HACC was accomplished through treatments with α-lipoic acid and 4-diethylaminobenzaldehyde to give the new derivatives **3l** (DS: 51–86%) and **3m**. In an alternate approach to the aforementioned procedure, the researchers initially established a Schiff base of CS, followed by a reaction with glycidyl trimethyl ammonium chloride, yielding *O*-quaternary ammonium *N*-benzylidene CS. To expand the scope of chemical modifications, *O*-quaternary ammonium *N*-acyl thiourea CS (**3n**; DS: 81–93%) was synthesized through the incorporation of ammonium thiocyanate and chloroacetyl chloride into the reaction sequence [108].

**Scheme 6:**
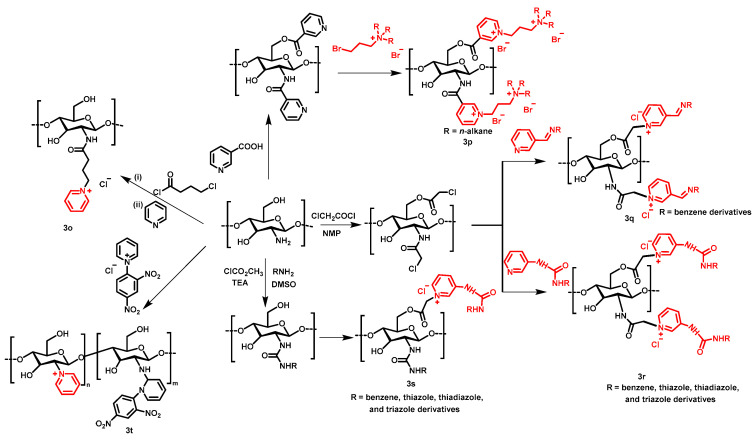
Synthesis of chitosan derivatives bearing pyridinium salt. The red color represents the functional group of pyridinium salt.

Another category of quaternary ammonium salt is the pyridinium salt group, which acts as a cationic surfactant, is soluble in water [87,109], and exhibits noteworthy antimicrobial and antifungal activities [110,111]. Pyridine functions as a nucleophile, capable of replacing the alkyl halide to synthesize the quaternary pyridinium compound [112]. Typically, CS undergoes modification through a reaction with either the halide group or the pyridine group, enabling subsequent functionalization with pyridine salts (Scheme 6) [113]. Huang et al. grafted pyridine to *N*-chlorobutyryl CS, resulting in the formation of pyridinium CS salt (**3o**, DS: 38%) [114]. *N*-chlorobutyryl CS was prepared from the reaction of CS and 4-chlorobutyryl chloride via acylation. Recently, Tan et al. synthesized CS-bearing pyridinium salt along with quaternary ammonium salt (DS: 35–97%) (**3p**) through a treatment between CS and narcotic acid, followed by a reaction with bromopropyl trialkyl ammonium bromides [115]. In another study, Guo et al. also introduced CS derivatives that contain pyridine-bearing Schiff bases (**3q**, DS: 22.7–50%) [116] and heterocyclic scaffolds along with thiourea (**3r**, **3s**; DS: 21–91%, 18–88%) [98,117]. In contrast with these conventional approaches, the Zincke reaction has been explored for the direct transformation of primary amines into *N*-aryl pyridinium salt [118]. This process typically involves the use of *N*-(2,4-dinitrophenyl)-pyridinium chloride, commonly known as Zincke salt, as the reagent, which follows an SN(ANRORC) mechanism involving nucleophilic substitution addition, ring opening, and subsequent ring closure [119]. In this respect, the CS amino group at the C2 position was directly converted to CS pyridinium salt (**3t**) via the Zincke reaction [120,121].

**Scheme 7:**
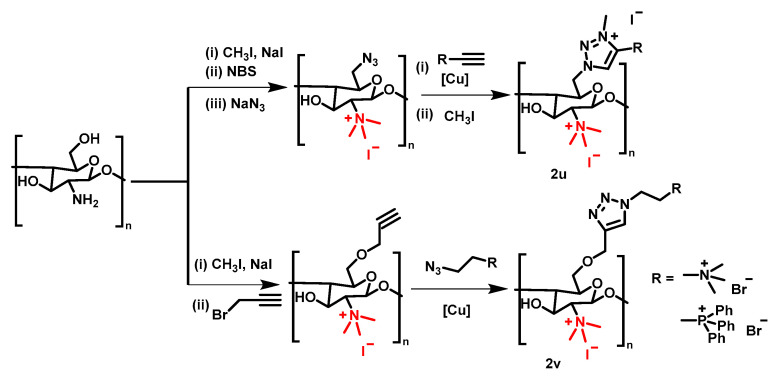
Synthesis of quaternary ammonium chitosan derivatives followed by click chemistry. The red color represents the functional group of quaternary ammonium salt.

The application of click chemistry has demonstrated significant utility in introducing quaternary ammonium salts into CS molecules, primarily due to its notable benefits of high efficiency and selectivity [122]. For instance, the initial step involved the development of *N*-trimethyl ammonium CS, which also served as a C2-NH_2_ protection group. Then, the reaction was followed by either azidation [123] or *O*-acetylation [124,125] to proceed with the cycloaddition reaction of alkyne-azides, resulting in the integration of water soluble triazole substituents with quaternary ammonium CS (**2u**, **2v**; DS: 72–83%, 28–92%, 53–92%), which exhibited antifungal and antioxidant properties (Scheme 7).

### 3.3. Guanidine Chitosan Derivatives

**Scheme 8:**
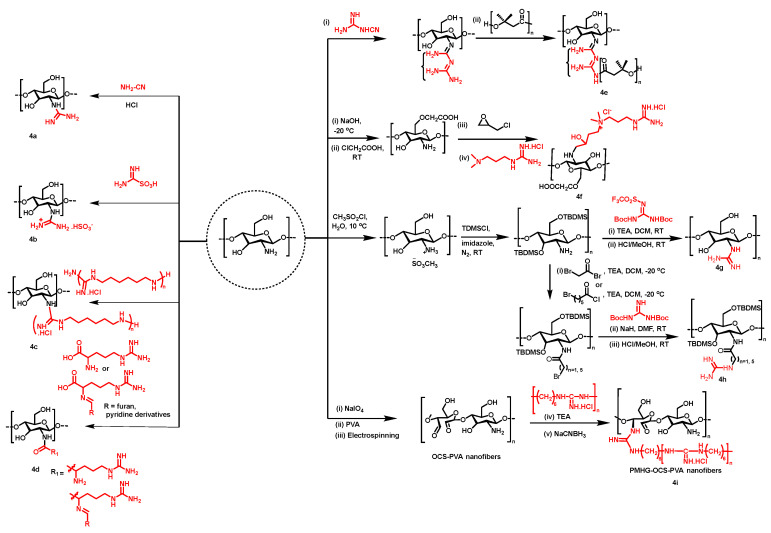
Synthetic strategy for guanidine chitosan derivatives. The red color represents the functional group of guanidine.

Guanidines have demonstrated a significant interest as a valuable functional group incorporated with multiple drugs for various therapeutic applications and biological activities, such as antimicrobial, antifungal, antimalarial, and others [126]. From a chemical perspective, guanidines function as the nitrogenous analog of carbonic acid, behaving as strong organic bases with higher pKa values. However, by introducing the appropriate substituents, such as CS, onto the nitrogen atom, the basicity of guanidine can be adjusted, and it is highly soluble in neutral aqueous solutions by disrupting hydrogen bonding and hydrophobic effects in polymers, resulting in the remarkable versatility of guanidine motif-bearing scaffolds [55,127]. Water-soluble CS-bearing guanidines (**4a**, **4b**; DS: 8.4–14.2%, 14–30%) are commonly synthesized through the reaction of cyanimide or aminoiminomethanesulfonic acid with CS in the presence of hydrochloric acid (Scheme 8) [128,129]. In the alternative pathway, the microwave-assisted grafting of guanidine oligomers with CS was also investigated, and the thus-formed CS guanidine (**4c**) in various DSs was utilized for the production of hygiene paper products [130]. Moreover, the main composition of the peptide, L-arginine, or L-arginine Schiff base, was also coupled with CS in a basic medium to develop biomimetic CS derivatives (**4d**; DS: 6–61.5%) for the improvement of the permeable capabilities of cell-penetrating peptides, wound healing, and antimicrobial and antioxidant activities [131,132,133,134,135].

Furthermore, CS–guanidine derivatives incorporating additional moieties such as poly (3-hydroxy butyrate) (**4e**) or quaternized carboxymethyl (**4f**; DS: 16–73%) were also synthesized (Scheme 8) [136,137]. In this process, cyanoguanidine or cyanimide functioned as the source of the guanidine. However, the reactions involved multiple sequential steps of the nucleophilic reaction of poly(3-hydroxybutyrate) or epichlorohydrin. Another approach for synthesizing guanidinylated CS derivatives involved the utilization of protective groups like tertiarybutyl dimethylsilyl (TBDMS) and tertiarybutyloxycarbonyl (Boc). These protective groups effectively overcome the limitation of selective modification of the amino group with a high degree of substitution. In the course of the reaction, the TBDMS group was initially introduced to the hydroxyl group of CS possessing a free amino group, enabling the successful synthesis of a 100% guanidinium substitution of the amino group, thereby yielding the desired guanidinium CS derivatives (**4g**, **4h**; DS: 13–100%) [138]. In the exploration of diverse derivatives, the Mingshan Li group functionalized the CS nanofiber membrane with the polyhexamethylene guanidine group (**4i**). The synthesis process included the oxidation of CS in the presence of sodium periodate (NaIO_4_), electrospinning with polyvinyl alcohol (PVA), and functionalization with polyhexamethylene guanidine [139]. Thus, the formed nanofiber membrane exhibited not only antibacterial activities but also high adsorption capacities of Cu (II) and Congo red.

**Scheme 9:**
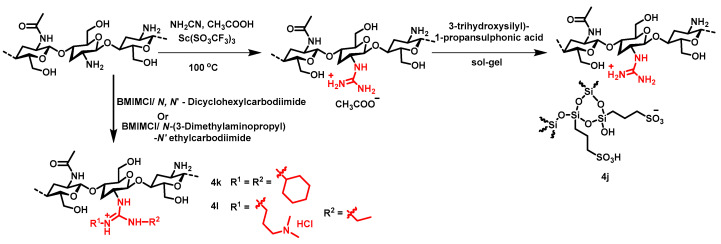
Synthetic strategy of guanidine chitosan salt derivatives. The red color represents the functional group of guanidine.

**Scheme 10:**
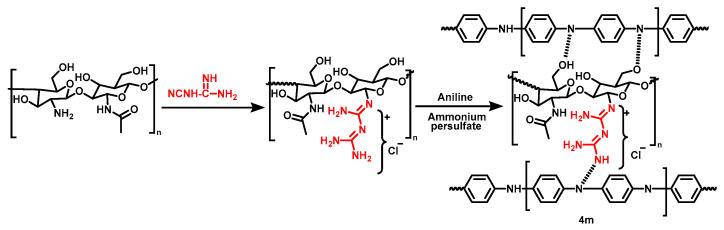
Synthetic strategy of biguanidine/polyaniline composite. The red color represents the functional group of guanidine.

In contrast to the aforementioned method, Peter Hesemann et al. employed a different approach to produce an *N*-guanidinium CS derivative. This was carried out in the presence of scandium (III) triflate and acetic acid, followed by a subsequent sol–gel reaction utilizing 3-(trihydroxysilyl)-1-propanesulphonic acid to synthesize a guanidinium CS silica hybrid material (**4j**) (Scheme 9) [140]. This material demonstrated remarkable effectiveness as an adsorbent for removing dyes from wastewater. On the other hand, the same group investigated the reaction between CS and carbodiimides in ionic liquid media in the presence of 1-butyl-3-methylimidazolium chloride (BMIM Cl) to produce *N*-guanidinium CS (*N*,*N*′-dicyclohexyl) chloride (**4k**) and *N*-guanidinium CS (*N*-(3-dimethylaminopropyl)-*N*′-ethyl hydrochloride) chloride (**4l**), showing significant amicrobial properties [141]. Similarly, the Sedghi group devised a semi-conductive water-soluble biguanidine/polyaniline composite (**4m**; DS: 18.6%) through the reaction of CS with cyanoguanidine followed by aniline incorporation (Scheme 10). Subsequently, the composite was further introduced into a self-healing waterborne polyurethane system, with shape-memory effect properties suitable for bone tissue engineering [142].

### 3.4. Heterocyclic Chitosan Derivatives via Click Chemistry or N-Functionalized Reaction

**Scheme 11:**
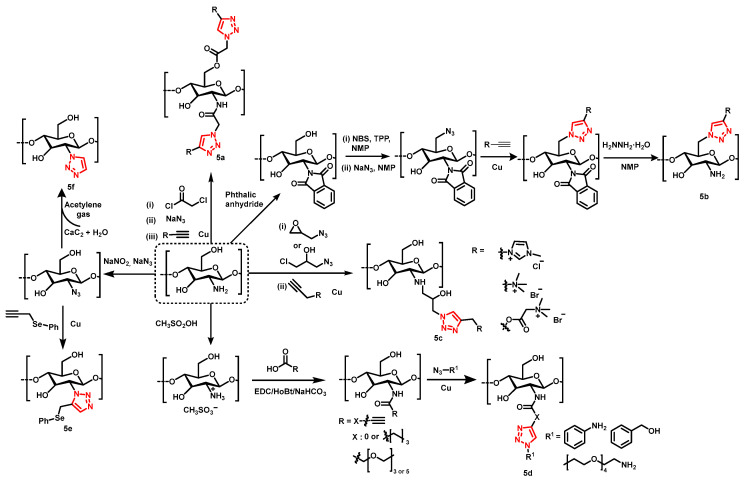
Synthetic strategy of heterocyclic chitosan derivatives through click chemistry. The red color represents the newly constructed triazole moiety.

Copper-mediated click chemistry has emerged as a versatile approach for synthesizing triazole moieties that involve the cycloaddition reaction between an azide and terminal alkyne, allowing for the incorporation of diverse bioactive functional groups into biomacromolecule backbones [143,144]. Furthermore, this strategy serves as a stable antimicrobial pharmacophore, displaying its dual significance [145]. In this regard, CS has been widely functionalized with heterocyclic scaffolds, such as substituted triazole groups formed through click reactions, and demonstrated significant antimicrobial and antifungal activities [49,124,146,147,148,149,150,151]. The primary advantage of incorporating triazole, a polar moiety, is its excellent water solubility, which imparts hydrophilic properties to the resulting CS derivatives bearing triazole groups [146,147,148,149,150,151]. CS has been modified by introducing an azido group or alkyne group into the 2-*N* or 6-*O* position for the click reaction (Scheme 11). The Li group introduced the azido group at both active positions of CS through the reaction of chloroacetyl chloride, followed by sodium azide, and subsequently reacted with prop-2-yn-1-yl nicotinate in the presence of copper sulfate to achieve antifungal (1,2,3-triazol-4-yl)methyl nicotinate CS (**5a**) [151]. The phthalolyl group was utilized for the protection of the 2-NH_2_ group, facilitated the 6-*O* position for azidation through stepwise bromination and azidation reactions, and was followed by the 3+2 cycloaddition reaction to provide diverse CS derivatives with triazoles (**5b**) [49,152]. Alternatively, another approach involved azidation through a nucleophilic NH_2_ reaction with compounds like 1-azido-3-chloro-2-propanol or 2(azidomethyl) oxiran.

Subsequent reactions with substituted acetylene in the presence of a Cu catalyst also led to the triazole-functionalized CS (**5c**) [153,154]. In addition, alkylation on the 2-*N* position through CS mesylate salt [155] or direct *N*-azidation in the presence of sodium nitrite and sodium azide [156,157] also enabled the click reactions to synthesize CS triazoles (**5d**, **5e**, **5f**).

**Scheme 12:**
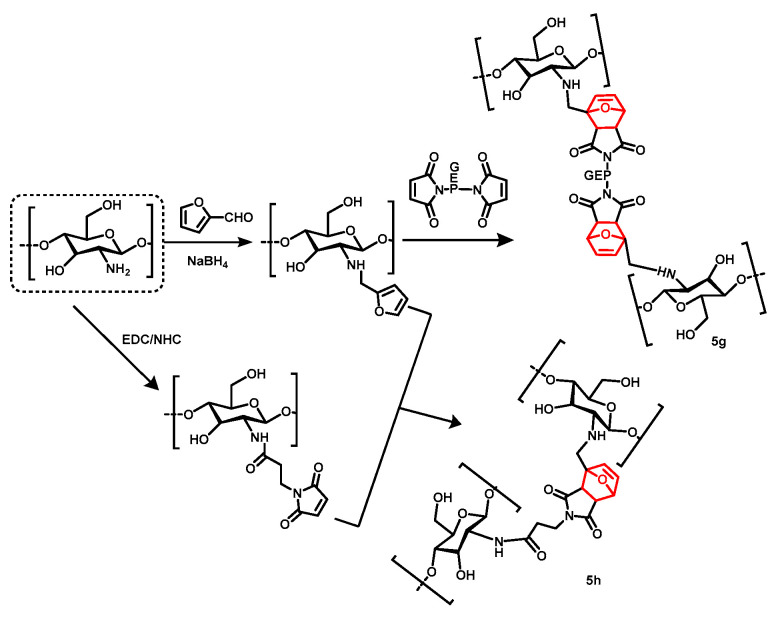
Synthetic strategy of furan-modified chitosan derivatives through Diels–Alder click reaction. The red color represents the creation of new bonds through Diels–Alder click reaction.

**Scheme 13:**
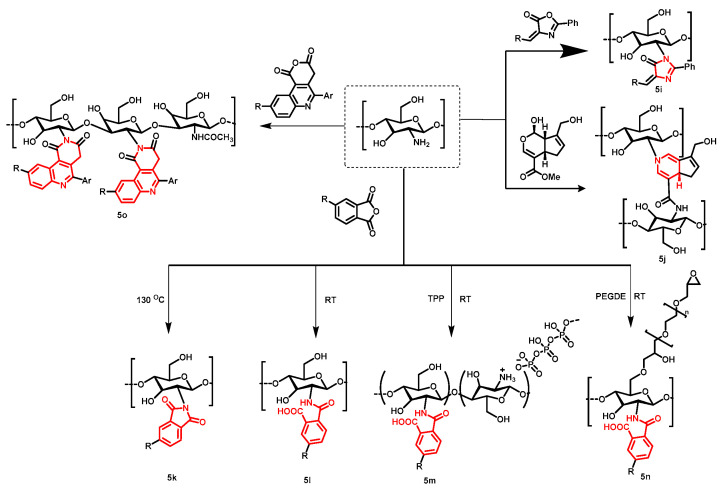
Synthetic strategy of heterocyclic chitosan derivatives through *N*-functionalized reaction. The red color represents the addition of new heterocyclic moiety through direct *N*-functionalization reaction.

In a further study of CS-based heterocycles, the researchers developed cross-linked CS-based hydrogels by using Diels–Alder reactions (Scheme 12). Furan-modified CS, which was obtained via the reaction of furfural with CS, was then subjected to the Diels–Alder click reaction with poly(ethylene)glycol-maleimide derivatives or pre-synthesized maleimide-functionalized CS in acidic conditions to prepare the hydrogels (**5g**, **5h**; DS: 21–31%) with the capability of controlled drug release [158,159]. Moreover, the researchers have also constructed CS azalactone gel (**5i**) or genepin-crosslinked CS hydrogel (**5j**) through the direct substitution of oxygen with the nitrogen of the NH_2_ group of CS (Scheme 13) [160,161]. In another approach for attaching the heterocycles to CS, the direct reaction of isobenzofurandione or pyrano-quinolinedione derivatives with CS led to the CS-heterocycles **5k**–**5n** (DS: 7–18%) and **5o** (DS: 34.79–92.05%) through either the direct interaction with the NH_2_ group or the ring opening of benzofurandiones [162,163].

## 4. Applications of Chitosan Derivatives

### 4.1. Antimicrobial Actions of Chitosan

CS has garnered extensive attention across a wide spectrum of applications, ranging from its role as an antimicrobial agent to its utility as a drug carrier. Numerous research investigations have sought to unveil CS’s antimicrobial attributes against a diverse array of microorganisms, including bacteria, fungi, yeast, and algae, primarily due to its biocompatible, biodegradable, and non-toxic properties [20,164]. The antimicrobial effects of CS are rooted in its physicochemical characteristics, which encompass the degree of deacetylation, its structural advantage in possessing reactive hydroxyl groups at the C-3 and C-6 positions, environmental conditions, and the specific microorganism in question [164]. The native CS, chitin, exhibits limited antimicrobial efficacy due to the presence of acetylated repeating units, which restrain the exposure of free amino groups. Furthermore, it is highly hydrophobic in nature because of the strong intra- and intermolecular hydrogen bonding [165]. On the other hand, CS-bearing repeating units of β-(1 → 4)-2-amino-2-deoxy-β-D-glucose offer primary amine functionality, typically achieved through the deacetylation process. A higher DD in the CS during the deacetylation process signifies an increased presence of free amino groups. These amino groups can interact with microbial cell surfaces through electrostatic attraction. Additionally, CS’s solubility in acidic water allows it to form cationic polyelectrolytes, facilitating its easy interaction with microbial cells and leading to cell lysis and cell death [166]. High-molecular-weight and low-molecular-weight CS exhibit distinct actions against microbes. High-molecular-weight CS’s antimicrobial activity primarily resides in the extracellular compartment, as it cannot penetrate the cell wall. Here, it operates by altering cell permeability, resulting in a reduced nutrient uptake from the extracellular milieu and a diminished availability of essential metals due to chelation. In contrast, low-molecular-weight CS displays the capability to exert both extracellular and intracellular effects. This translates to alterations in mitochondrial function and consequential impacts on RNA and protein synthesis [167,168].

**Figure 1:**
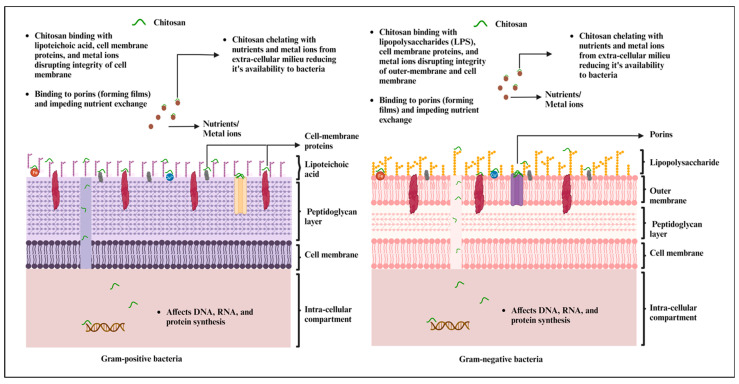
Mechanism of antimicrobial effects of chitosan and chitosan-based materials against Gram-positive and Gram-negative bacteria (created with BioRender.com, accessed on 27 September 2023).

CS has undergone extensive testing against both Gram-positive and Gram-negative bacteria for antibacterial activity (Figure 1, Table 2). However, the precise mechanism of action remains incompletely understood. Most studies underscore CS’s polycationic nature as a key contributor to its antibacterial efficacy [169]. Furthermore, its interactions with the bacterial cell wall are also dependent on the pH of the environment. When the environmental pH falls below CS’s pKa, electrostatic interactions occur between the polycationic CS and the anionic components of the bacterial cell wall. These components include lipopolysaccharides in the Gram-negative cell wall and certain proteins on the cell surface of bacteria. These interactions lead to the exertion of antibacterial effects [22,169]. Interestingly, the extent of electrostatic interactions is largely influenced by the number of amino groups linked to C-2 on the CS backbone. A higher number of amino groups results in more positively charged sites, enhancing the interaction with the carboxyl-negative charges on the bacterial cell wall [170].

Conversely, when the environmental pH exceeds the pKa, electrostatic interactions weaken, and chelating and hydrophobic effects come into play for antibacterial activity [169,170]. When chelation effects outweigh electrostatic forces, metal ions like Fe^2+^, Cu^2+^, and Zn^2+^ present on the bacterial surface can be chelated by CS’s amino groups [164,169,171]. Many of these divalent cations play a vital role in stabilizing bacterial cell membranes through their interactions with phosphate groups in teichoic acids and lipopolysaccharides (LPSs) in Gram-positive and Gram-negative bacteria, respectively [172,173]. Variations in susceptibility to CS exist between Gram-positive and Gram-negative bacteria due to marked differences in their cell wall compositions. Gram-positive bacterial cell walls consist of peptidoglycan, teichoic acids, and lipoteichoic acids [174,175]. These components contribute to high-density negative charges within the cell wall, hindering ion movement across the membrane. Positively charged CS can interact with these negatively charged teichoic acids in peptidoglycans, leading to cell membrane damage and the release of intracellular constituents [22,176]. Studies have also demonstrated that interactions with CS can result in peptidoglycan hydrolysis, intensifying electrostatic interactions, and enhancing antibacterial activity [177].

In Gram-negative bacteria, CS targets negatively charged lipopolysaccharides, causing the disruption of the outer membrane, the penetration of the cell membrane, and ultimately, bacterial death [178,179]. High-molecular-weight CS can form a dense polymer film on the bacterial cell surface, obstructing porins and impeding nutrient exchange, which ultimately leads to bacterial cell death [178,180]. Evidence of this is observed in scanning electron microscope studies, which revealed vesicle-like structures on the outer membrane of CS-treated *Escherichia coli* (*E. coli*) and *Salmonella typhimurium* (*S. typhimurium*) [181]. On the contrary, low-molecular-weight CS can penetrate bacterial cells, affecting DNA, RNA, and protein synthesis [20]. Studies have shown that when *E. coli* has been exposed to oleoyl-CS nanoparticles, the nanoparticles bind to DNA and RNA and influence the electrophoretic mobility of both nucleic acids [171]. CS and its derivatives have been extensively explored for their antimicrobial properties against various pathogens, offering diverse mechanisms of action [182]. Thioether CS oligosaccharides and CS–silver nanocomposites have been explored for their synergistic antimicrobial effects against multiple Gram-positive and Gram-negative bacteria [183,184]. Furthermore, another study reported the increased antibacterial activity of *N*-(2-ethylamino)-CS and *N*-2(2,6-diaminohexanamide)-CS polymers, attributed to the presence of amino groups and their hydrophobic interactions [185]. Similar studies have introduced CS Schiff bases with aromatic substitutions, which exhibit enhanced interactions with bacterial cell components [186]. CS Schiff bases were developed to potentially interact with specific components in both Gram-positive and Gram-negative bacteria, exerting antibacterial action [187]. The synthesis and antimicrobial properties of *N*-guanidinium CS derivatives were also investigated, highlighting their permeability-enhancing effects through adsorption and intracellular binding [141].

CS nanoparticles, when loaded with antibiotics, peptides, and proteins, have displayed promising results in possessing antibacterial effects and disrupting biofilm formation by bacteria. For instance, ceftriaxone sodium encapsulated within CS nanoparticles has exhibited heightened intracellular antibacterial efficacy against *S. typhimurium* [188]. Cefazolin-loaded CS nanoparticles have demonstrated enhanced antibacterial activity, particularly against multi-drug-resistant Gram-negative bacteria [189]. Meanwhile, tetracycline-loaded *O*-carboxymethyl CS nanoparticles have shown the potential to augment the action of antibiotics against *Staphylococcus Aureus* (*S. aureus*) infections [190]. Similarly, CS-based systems have been widely used for assessing their potential as carriers for probiotics or beneficial bacteria that can be used for controlling pathogenic bacterial infections. In one such study, our group demonstrated that CS–alginate nanoparticles carrying *E. coli* Nissle effectively inhibited *Campylobacter jejuni* growth in vitro [191].

Within the realm of CS metal nanocomposites, silver-based variants have received substantial attention. Several studies have revealed their effectiveness against a spectrum of bacteria, including *S. aureus*, *E. coli*, and *S. typhimurium* [190,192,193]. Furthermore, CS-based systems have been evaluated for their impact on biofilms. Anti-biofilm properties have been explored using preparations such as CS–streptomycin conjugates and CS–gold nanoparticles against various Gram-positive and Gram-negative bacteria. These studies have revealed that CS conjugation enhances the drug’s ability to disrupt biofilms by improving its penetration and contact with the bacterial surface [194]. Due to these antimicrobial properties, CS-based systems have undergone extensive investigation for their potential applications in wound healing, drug delivery carriers, and various biomedical applications.

The utilization of CS as an antimicrobial agent presents a dynamic landscape of potential research avenues and practical applications. Nanoparticle engineering is poised to harness CS’s inherent properties for the development of finely tuned nanoparticles, enabling precise, targeted antibacterial delivery with a focus on enhanced efficacy. Synergy exploration within combination therapies involving CS and other antimicrobial agents unveils an exciting frontier in the fight against antibiotic-resistant pathogens. In-depth investigations into the intricate molecular interactions governing CS’s biofilm disruption capabilities hold significant promise for novel strategies for combating persistent biofilm-associated infections. Concurrently, rigorous assessments of CS’s biocompatibility and toxicity profiles are vital, ensuring the safe integration of CS-based therapies into clinical practice. The vast potential of CS derivatives including CS oligomers and quaternized CS beckons, as they offer tailored solutions to specific antibacterial challenges. Elucidating the precise mechanisms underpinning CS’s antibacterial action, encompassing cell membrane interactions and resistance mechanisms, as well as understanding the host immune responses to CS, not only advances fundamental knowledge but also guides the rational design of more potent therapies in the future.

**Table 2:** Application of chitosan and its derivative for antibacterial activities.

CS Derivative/Preparations	Bacteria	Assay	Effect/Mechanism of Action	References
CS with benzoimidazolyl-thiadiazole	*S. aureus*, *B. subtilis*, *E. coli*, *P. aeruginosa*, *C. albicans*	Agar well diffusion method	Presence of polar groups, sulfur, and nitrogen, increases the solubility and electrostatic attraction between polymer and bacterial cell wall and increases cell death	[70]
Thiadiazole CS derivative	*E. coli*, *P. aeruginosa*, *B. subtilis*, *S. aureus*	Agar well diffusion method	Increase solubility due to cationic groups contributing to improved antibacterial activity	[71]
1,3,4-thiadiazole modified CS	*E. coli*, *P. aeruginosa*, *B. subtilis*, *S. aureus*	Agar well diffusion method	Increase solubility due to hydrophilic thiadiazole derivatives	[72]
Thymine-modified CS	*P. aeruginosa*, *A. baumannii*, *S. aureus*, *E. coli*, MRSA	96-well plate microdilution method, scanning electron microscopy	Increase ζ-potential of the positively charged thiamine-modified CS derivatives; higher positive electric density; enhance electrostatic interaction with the negatively charged bacterial membrane	[74]
CS linked with diphenyl pyrazole with succinic anhydride	*B. subtilis*, *S. aureus*, *P. aeruginosa*	Agar well diffusion method	Polycationic nature of CS and presence of amino group in pyrazole; penetration of CS into nucleus, blocking binding sites of RNA to DNA, inhibiting synthesis of cell wall proteins	[75]
CS coupled with 4-((5, 5-dimethyl-3-oxocyclohex-1-en-1-yl) amino) benzene-sulfonamide (CS Schiff base)	*E. coli*, *S. aureus*	Visible spectroscopy analysis, MIC assay	Increase hydrophobicity of CS Schiff base, improving interaction with peptidoglycan	[78]
CS with heteroaryl pyrazole derivatives (CS Schiff base)	*E. coli*, *K. pneumonia*, *S. aureus*, *S. mutans*	Agar well diffusion method	Functional modification with pyrazole ring bearing pyridyl moiety enhanced antibacterial effect	[80]
CS with formyl pyrazole derivatives (CS/pyrazole Schiff base)	*S. aureus*, *B. cereus*, *E. coli*	Agar well diffusion method	Presence of furan ring and nitro groups; enhance entry of CS to nucleus and interaction with RNA/DNA	[81]
CS with indole-3-carboxaldehyde and 4-dimethylaminobenzaldehyde (phenolic CS Schiff bases)	*S. aureus*, *B. cereus*, *E. coli*, *P. aeruginosa*, *S*. spp.	Agar well diffusion method	Interaction with bacterial cell membrane and disruption of cell wall integrity	[82]
Chitooligosaccharide-niacin acid conjugate	*S. aureus*, *E. coli*, *V. harveyi*	Broth dilution assay	Increase lipophilicity, hydrophobic interaction of the trialkyl chain, increased interaction of quaternary ammonium salts and lipid structure of bacterial cell membrane	[115]
Pyridine-4-aldehyde Schiff bases grafted chloracetyl CS oligosaccharide derivatives	*S. aureus*, *E. coli*	Agar well diffusion method	Increase positive charge of the derivative and interaction with bacterial cell membrane	[116]
L-arginine Schiff bases acylated CS derivatives	*B. cinerea*, *S. aureus*, *E. coli*	Plate colony counting method	Free positive charge and guanidine carried by CS combines with the negatively charged components of the bacterial cell wall	[135]
*N*-guanidinium CS acetate, *N*-guanidinium CS chloride	*E. coli*, *P. aeruginosa*, *S. aureus*, *B. subtilis*	Turbidimetric method	Cationic groups enhance the permeability via adsorption followed binding to intracellular constituents	[141]
Chitotriazolan (poly(β(1-4)-2-(1H-1,2,3-triazol-1-yl)-2-deoxy-D-glucose))	*S. aureus*, *E. coli*	96-well plate microdilution method	Loss of integrity to bacterial cell wall	[146]
Calcium–CS–triazole nanocomplex	*E. coli*, *B*. *subtilis*	Agar well diffusion method	Enhance antibacterial activity contributed by triazole moiety	[157]
CS hydrogel containing hydroxypropyl methylcellulose (HPMC)	*S. aureus*, *P. aeruginosa*	Biofilm assay/confocal scanning laser microscopy	Increase adhesiveness and penetration into biofilm and disruption	[182]
Thioether CS oligosaccharide (CS oligosaccharide coupled with 3-bromopropene and tiopronin)	*S. aureus*, *B. subtilis*, *L. monocytogenes*, *E. coli*, *P. aeruginosa*	MIC and MBC determination, cytotoxicity evaluation, antioxidant activity assays	Enhance antimicrobial capability due to carboxyl groups possessing positive charges, which bind to negatively charged cell membranes. Increased iron chelation from tiopronin.	[183]
Biogenic CS–silver nanocomposite	*S. aureus*, *P. aeruginosa*, *S*. spp., *E*. spp., *S*. spp., *Shigella* spp.	Well diffusion technique for antimicrobial susceptibility	Synergistic effect of CS and silver; greater adsorption onto the surface of bacterial cells and can easily penetrate the bacterial cell wall causing cell death	[184]
*N*-(2-ethylamino)-CS and *N*-2(2,6-diaminohexanamide)-CS polymers	*S. aureus*	Bacterial growth inhibition, cytocompatibility studies	Presence of the amino groups: hydrophobic interaction with the bacterial wall is responsible for the enhanced activity	[185]
CS Schiff base (CS with 2,4,6-trimethoxybenzaldehyde)	*S. aureus*, *E. coli*, *P. aeruginosa*, *K. pneumonia*, *S. haemolyticus*	Turbidity assay	Enhance interaction of CS with peptidoglycan and plasma membrane due to aromatic substitution	[186]
CS coupled with cyclohexanone and 2-*N*-methyl pyrrolidone (CS Schiff base)	*S. aureus*, *E. coli*, *P. aeruginosa*, *B. cereus*	Agar well diffusion assay, MIC determination, bactericidal studies	Interaction with teichoic and lipoteichoic acid in Gram-positive bacteria and with O-specific side chain of LPS of Gram-negative bacteria	[187]
CS with immobilized ZnO nanoparticles	*E. coli*, *S. aureus*	Agar well diffusion method	Antibacterial activities	[195]
Low-molecular-weight CS	*E. coli DH5α*, *P. putida* F1, *L. lactis* IO-1, *B. subtilis* 168	Zeta potential measurement, contact angle measurement	Alteration of cell surface charge, cell surface hydrophobicity	[196]
CS nanoparticles	*P*. spp.	Disk diffusion test, antibiofilm assay	Compromise structural integrity of the biofilms, heterogeneous destruction of the biofilm matrix	[197]
Thiazolium CS (Quaternary CS)	*L. innocua*, *S. epidermidis*, *S. aureus*, MRSA	Broth dilution method	Combine antibacterial effect of thiazolium and CS	[198]
CS covalently bounded to isocyanate terminated quaternary ammonium salt and sulfopropylbetaine	*E. coli*, *S.aureus*	Real-time turbidity monitoring via automated turbidimetric system	NH_2_ group of the derivative binds with Mg^2+^ and Ca^2+^ in bacterial outer membrane	[199]

Note: *S. aureus*: *Staphylococcus aureus*, *B. subtilis*: *Bacillus subtilis*, *E. coli*: *Escherichia coli*, *P. aeruginosa*: *Pseudomonas aeruginosa*, *C. albicans*: *Candida albicans*, *A. baumannii*: *Acinetobacter baumannii*, *MRSA*: *Methicillin-resistant Staphylococcus aureus*, *K. pneumonia*: *Klebsiella pneumonia*, *S. mutans*: *Streptococcus mutans*, *B. cereus*: *Bacillus cereus*, *S*. spp.: *Salmonella* spp., *V. harveyi*: *Vibrio harveyi*, *B. cinerea*: *Botrytis cinerea*, *L. monocytogenes*: *Listeria monocytogenes*, *E*. spp.: *Enterococcus* spp., *S. haemolyticus*: *Staphylococcus haemolyticus*, *P. putida* F1: *Pseudomonas putida* F1, *E. coli DH5α*: *Escherichia coli DH5α*, *L. lactis* IO-1: *Lactococcus lactis* IO-1, *B. subtilis* 168: *Bacillus subtilis* 168, *L. innocua*: *Listeria innocua*, *S. epidermidis*: *Staphylococcus epidermidis*.

### 4.2. Chitosan Derivatives as Drug or Vaccine Delivery System

**Figure 2:**
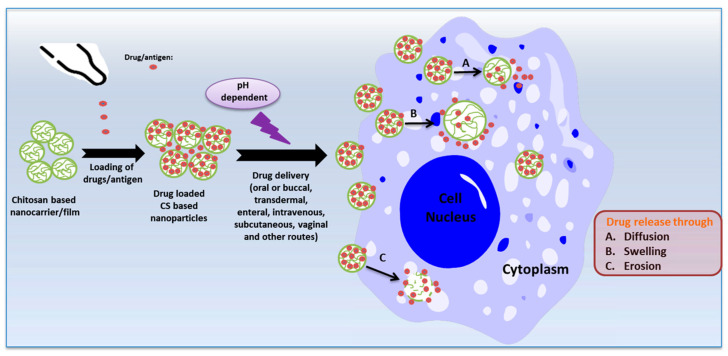
The mechanisms of release of drugs/antigens from chitosan nanoparticles (created with Microsoft Power Point 2010 and Chemdraw 19.1).

Controlled drug or vaccine release is highly emphasized as a strategy for controlling disease pathologies, achieved through the targeted accumulation of therapeutic compounds at the site of the ailment or the utilization of inventive therapeutic agents to control infectious pathogens [200,201]. Drug encapsulation and dosage reduction emerge as the most convenient methods for achieving controlled drug release [202]. CS and its derivative-based nanoparticles and films have received considerable attention as highly suitable candidates for drug or antigen carriers due to their mucoadhesive properties, pH sensitivity, and biocompatibility. The release of drugs/antigens from CS nanoparticles in biological fluids occurs through diffusion, erosion, or swelling mechanisms [203] (Figure 2). Additionally, their distinct antimicrobial activities collaboratively synergize with drugs to enhance their antimicrobial effectiveness [204].

The recent applications of CS-based nanomaterials as drug carriers against infectious diseases are summarized in Table 3 [205,206,207,208,209,210,211,212,213,214,215,216,217,218]. For example, the nasal delivery of hesperidin-loaded CS nanoparticles exhibited enhanced cellular uptake within inflammatory microenvironments and effectively suppressed cytokine storm syndrome and acute lung injury [205]. Meanwhile, CS biguanidine nanoparticles displayed remarkable inhibition against various *Mycobacterium tuberculosis* strains, including sensitive, multidrug-resistant (MDR), and extensively drug-resistant (XDR) types, surpassing the performance of the standard anti-tuberculosis drug, isoniazid [206]. Sawant et al. demonstrated that tuberculosis prevention could be achieved through the pulmonary delivery of rifampicin with improved solubility when encapsulated within octanoyl CS nanoparticles [207]. Additionally, CS-based materials proved effective in delivering various anti-infectious drugs and antibiotics, including carvacrol [208], doripenem [209], chlorhexidine [210], ciprofloxacin [211], dolutegravir [212], endolysin Cpl-1 [213], and amphotericin B [214]. These materials successfully inhibited the growth of pathogens such as *Salmonella enterica*, *Pseudomonas aeruginosa*, *Staphylococcus aureus*, *Staphylococcus epidermidis*, *Escherichia coli*, human immunodeficiency virus HIV, and fungal species. In the expanded application, the eradication of waterborne pathogens such as rotavirus and *Shigella* spp. was also investigated through CS-wrapped carbon nanotubes [215].

In highlighting the effort in the encapsulation process, the inactivated virus, RNA, proteins, and probiotics have also been capsulated in the CS derivatives, which serve as a nano-vaccine delivery system to increase the immune response against the pathogens [219,220,221,222,223,224,225]. As an instance, Tafaghodi et al. encapsulated the inactivated PR8 influenza virus within CS alginate and trimethyl CS nanoparticles which exhibited immunoadjuvant capabilities after nasal immunization, thereby stimulating a Th1-type immune response [219]. Inactivated dengue virus (DENV)-loaded CS nanoparticles displayed their high immunogenicity, inducing elevated levels of antibodies and T cells against DENV-2 in comparison with soluble DENV-2 immunogens [220]. CS-based nanoparticles, containing *Salmonella Enteritidis* outer membrane proteins and flagellin proteins, emerged as a mucosal vaccine, inducing specific immune responses and reducing cecal colonization in avian hosts [221]. Our group also demonstrated the microencapsulation of probiotic *E. coli* Nissle 1917 within the CS–alginate, which reduced *Campylobacter jejuni* colonization in infected chickens [222].

Due to their cationic nature and high endocytosis potential, CS and its derivatives are not only utilized for drug and vaccine delivery for infectious diseases but also find applications in addressing various other conditions, such as cancer, gastrointestinal disorders, pulmonary ailments, Alzheimer’s disease, and diabetes [226,227,228,229,230,231,232,233,234,235,236,237,238,239]. Table 4 provides a summary of drug delivery systems designed to treat a range of diseases utilizing various forms of CS and its derivatives. CS-based nanoparticles enhance the drug-targeting ability and improve the blood retention time through desorption, swelling, and erosion. For instance, a PEGylated CS nanoparticle was employed to deliver a chemotherapeutic agent such as doxorubicin (DOX) in combination with breast cancer-specific monoclonal antibodies to treat breast cancer [226]. Thiolated CS nanoparticles delivered vincristine by binding to leaky prostate tumor cells, stimulating the receptors of these cells, and facilitating their internalization through endocytosis [227]. In another approach to the drug delivery system, the formulation of CS-based films and patches was employed in the ocular and transdermal delivery of the drugs brimonidine tartrate (BT) and tizanidine, respectively [231,232]. The CS-BT film significantly improved the transport of BT across the cornea, offering a potential alternative eye drop in the future, whereas the transdermal delivery of tizanidine facilitated efficient drug permeation through the skin, maintaining a therapeutic concentration. Moreover, CS and its derivatives have been fabricated through the crosslinking and polymerization methods to prepare hydrogels that have demonstrated a controlled, painless, and biocompatible transdermal release of therapeutic ingredients [233]. In an effort to find suitable carriers, CS-based micelles and liposomes have emerged as innovative tools to address the challenges associated with drug administration, especially the issues of low water solubility and limited drug permeability [234,235,236,237,238]. For example, the amphiphilic colloidal structures, especially CS-based micelles, including the CS-bearing thiourea group, incorporated with gold nanorods and carboxymethyl CS with a vitamin E succinate nano-micellar system (*O*-CMCTS-VES), effectively facilitated the release of drugs like paclitaxel and DOX for antitumor activities [234,235]. In addition, CS derivatives were employed to encapsulate liposomes, which possess minimal toxicity and cell-like membranes, along with drugs like resveratrol, aptamer-functionalized 5-fluorouracil, photosensitizer HPPH, and hypoxia-activated prodrug TH-302, enhancing the efficient targeted delivery of these drugs [236,237,238].

The drug-release characteristics of CS nanoparticles exhibit intricate responsiveness to pH alterations, rendering them a versatile tool for precision drug delivery. Research indicates that alterations in pH levels induce significant shifts in CS nanoparticle size and drug molecule release. In a study incorporating 5-fluorouracil (5-FU) within CS nanoparticles, it was observed that as the pH transitions from 3 to 5, there is a substantial increase in particle size, resulting in a continuous and rapid release of 5-FU. Conversely, when the pH exceeds 5, a notable reduction in particle size is evident, particularly at pH 7.4. This pH-responsive behavior can be attributed to the protonation of primary amino groups within the CS chain, leading to heightened electric density and increased repulsion forces between interconnected CS chains. Consequently, the surface density of protonated amino groups and the degree of protonization are adaptably responsive to shifts in solution pH [240]. CS nanoparticles undergo a reversible process of swelling and contraction, contingent upon the environmental pH, resulting in particle sizes fluctuating from approximately 450 nm to 150 nm [241]. The pH-sensitive surface charge reversal also facilitates enhanced cellular uptake and endolysosomal escape, extending blood circulation time, mitigating side effects, and optimizing drug delivery efficiency [242]. Such adaptability holds significant promise for targeted drug delivery, aligning with the varying pH levels found within the human body, which include gastric juice with a low pH of 1–1.5, the physiological environment exhibiting a pH of 7.4, the tumor extracellular microenvironment typically with a pH 6.5, and the pH within endosomes and lysosomes ranging from 5.0–6.2 to 4.0–5.0, respectively [242,243]. Moreover, studies highlight the enhancement of responsive properties through the integration of pH-sensitive molecules with CS, further streamlining drug delivery in response to environmental cues [244]. Additionally, investigations demonstrate that under acidic conditions (pH 3.5), there is a swift release of model drugs, while at slightly alkaline conditions (pH 7.4), the release rate is attenuated [245].

Despite the encouraging implications of CS and its derivatives as a drug or vaccine carrier system, they suffer from specific limitations. These limitations involve factors such as a restricted capacity to carry larger-sized drugs or vaccines, issues related to stability, the occurrence of burst drug release upon administration, limitations in targeting specific diseases, and the potential for an immune response in certain individuals. Nevertheless, ongoing research on the development of novel drug carriers that are more biodegradable, non-invasive, and have a high drug-loading capacity is actively employed to overcome these constraints. In the future, there is potential for designing CS derivative nanoparticles in a computational way that may not only be capable of their targeting efficacy but also have the ability to interact with proteins and signaling pathways. This system could potentially reduce the drug dosage requirements with minimal side effects and improve the patient’s condition.

### 4.3. Chitosan Derivatives in Plant Agriculture

**Figure 3:**
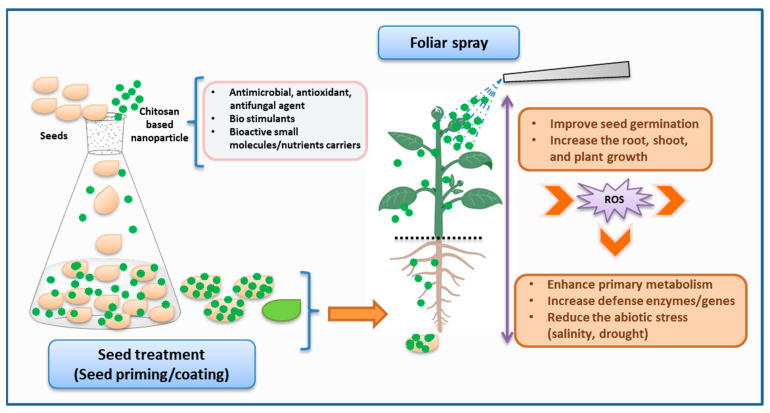
Schematic representation of the application of chitosan-based nanoparticles in plant agriculture (created with Microsoft PowerPoint 2010, Chemdraw 19.1, and BioRender.com, accessed on 27 September 2023).

CS and its derivatives have gained significant interest in the agriculture field, especially for their role in enhancing plant growth, stimulating root and shoot development, increasing crop yield, and supporting environmentally friendly farming practices (Figure 3) [246]. The cationic property of CS derivatives enables effective interaction with the negatively charged components of the bacterial cell membrane, resulting in bactericidal or bacteriostatic effects [247]. On the other hand, the chelating characteristic of CS also establishes it as a tremendous antifungal agent [248]. Similarly, it has also demonstrated its role as an elicitor by activating the defensive genes in plant disease control [249].

Considering the encouraging advantages, CS derivatives have been utilized in a wide range of crops, including vegetables, flowers, fruits, cereals, and medicinal plants, in various forms such as seed treatment, foliar spraying, coating fruits or vegetables, and delivering nutrients through nanomaterials [250] (Table 5). For example, Udayashankar group investigated the CS exogenous seed priming of cucumbers seeds, resulting in the inducement of phytohormone production and an increment of resistance against cucumber powdery mildew disease [251]. Recent studies demonstrated the use of CS through seed priming, seed soaking, or seedling application in mung bean [252], *Platycodon grandiflorus* [253], and *Carum copticum* L. [254] for reducing abiotic stress and increasing photosynthesis and antioxidant activity, resulting in efficient plant growth. Similarly, plant diseases such as root rot caused by *Fusarium solani* in fenugreek were also managed with seed treatment [255]. CS derivatives such as CS–ferulic acid, CS–caffeic acid conjugate, and carboxymethyl CS were explored for seed treatment or seed coating in cucumbers [256] or *Prunus davidiana* [257]. As a result, they either increased the antioxidant activity or enhanced the biomass, photosynthetic capacity, and absorption of phosphorus and potassium to improve plant growth. In further applications, CS nanoparticles have been significantly introduced in seed treatment [258]. For instance, Jogaiach et al. explored CS-derived nanoparticles in tomato seed treatment, which led to a remarkable increase in immune responses by accumulating defense enzymes and lignin to eradicate bacterial wilt disease [259]. CS incorporated with organic compounds or metals, such as garlic essential oil or silver-loaded nanoparticles, demonstrated its application in seed treatment on barley or chilies, which reduced the antifungal activities in plant growth [260,261].

CS and its derivatives, along with nanoparticles, were also employed as foliar sprays to reduce plant diseases, functioning as elicitors, plant protectors, or biostimulants [262,263]. For example, the foliar spraying of CS or its nanoparticles for tomatoes elicited an increment in shoot biomass and flower numbers, with reduced interaction with arbuscular mycorrhizal fungi (AMF) [264]. CS foliar spraying significantly reduced drought stress in bermudagrass by enhancing the turf quality, chlorophyll content, and leaf relative water content, and decreasing the level of electrolyte leakage, malonaldehyde, and hydrogen peroxide content [265]. In another study, the spray of CS derivatives such as CS lactate on *Ocimum basilicum* L. and *Melissa officinalis* L. accumulated the valuable phenolic group rosmarinic acid (RA) and increased the shoot biomass [266]. This showed the capability of herbal plants to produce functional foods. In addition, by using the CS–selenium nanoparticle, Skalicky et al. explored foliar spray with bitter melons, alleviating salt stress by enhancing antioxidant enzyme activity, elevating proline concentration, maintaining relative water content and K^+^, and reducing malonaldehyde and hydrogen peroxide levels, as well as sodium accumulation in plant tissues [267].

Despite the plethora of studies of CS applications demonstrating remarkable outcomes in enhancing plant growth, disease resistance, and stress tolerance, there always remains a requirement to determine the optimal techniques, dosage, and timing for its most effective use [246,268]. Through ongoing research, technological innovation, and eco-friendly techniques, CS has the capability to revolutionize harmless agricultural methods, which could pave the way for a more sustainable agricultural future.

**Table 5:** Chitosan and its derivatives for applications in plant agriculture.

CS or Its Derivatives	Form	Plant	Interference	References
CS	Seed priming	Cucumber	Upregulation of phytohormones; Resistance against cucumber powdery mildew disease.	[251]
CS	Seed priming	Mung bean	Alleviate water stress, increase antioxidant system, improve growth.	[252]
CS	Seed soaking	*P*. *grandiflorus*	Enhances plant growth, photosynthesis, resistance, yield, and quality.	[253]
CS	Seedling and callus culture	*C*. *copticum* L.	Improve plant tolerance to oxidative stress under salinity; Elevation of essential oils and antioxidant activity.	[254]
CS	Seed treatment	Fenugreek	Controlling the root rot disease by reducing *F. solani;* Increase seed germination.	[255]
CS– ferulic acid and CS– caffeic acid conjugate	Seed treatment	Cucumber	Increase plant growth; Antioxidant activity.	[256]
Carboxymethyl CS	Seed coating	*P*. *davidiana*	Increase the biomass and photosynthetic capacity; Promote the absorption of phosphorus and potassium; Decrease nitrogen uptake.	[257]
CS nanoparticle	Seed treatment	Tomato	Enhancing the immune response by accumulating the defense enzyme and lignin.	[259]
Garlic essential oil-loaded CS nanoparticle	Seedling	Barley	Antifungal activity; Alternative to tebuconazole as seed-dressing agent.	[260]
CS silver nanocomposites	Seed treatment	Chili	Antifungal activity against anthracnose pathogen.	[261]
CS and its nanoparticles	Foliar spray	Tomato	Enhance plant growth and flowering.	[264]
CS	Foliar spray	Bermudagrass	Improve drought tolerance.	[265]
CS lactate	Foliar spray	*O*. *basilicum* L. and *M*. *officinalis* L.	Enhance the accumulation of valuable phytochemicals.	[266]
CS–selenium nanoparticle	Foliar spray	Bitter melon	Increased salinity tolerance in bitter melon plants.	[267]

Note: *P. grandifloras*: *Platycodon grandifloras*, *C. copticum* L.: *Carum copticum* L., *P. davidiana*: *Prunus davidiana*, *O. basilicum* L.: *Ocimum basilicum* L., *M. officinalis* L.: *Melissa officinalis* L.

## 5. Conclusions and Future Outlooks

CS is considered a promising polysaccharide renowned for its extensive array of potential applications in the fields of biology, medicine, water treatment, the food industry, cosmetics, and biodegradable packaging. Certain constraints persist, such as a lower degree of deacetylation and elevated molecular weight, which directly result in poor water solubility in neutral aqueous solutions. The modification of CS through different strategic pathways is one perspective that not only improves the water solubility but also enhances the bioactivity of the final product. However, the CS-bearing long chain or hydrophobic scaffolds may diminish the water solubility; nevertheless, they can still be effectively incorporated into films, fibers, or polymeric materials to enhance the hydrophobicity and stability of the materials [269]. This review highlighted the recent methodologies for synthesizing CS derivatives and their notable uses in antimicrobial functions, nanomaterials employed in drug delivery systems, and applications within the agricultural sector. Recent progress in the development of CS derivatives has been achieved through various methods, such as forming Schiff bases with heterocyclic compounds, generating quaternary ammonium salts and guanidines, employing click chemistry, and using them in direct *N*-functionalization reactions. There are also critical challenges, including separation, purity, yield, and stability, to developing new CS-bearing compounds.

Exploring CS’s use as an antimicrobial agent unmasks a dynamic array of research possibilities, especially in the medical and agricultural fields. CS demonstrates significant bioactivity in combating both animal and plant pathogens. The research on nanoparticle engineering involving CS and its derivatives as drug carriers has significantly demonstrated the effective delivery of drugs or vaccines to target diseases in the pharmaceutical field. Antimicrobial dressings based on CS and its derivatives have received FDA approval [270,271]. However, new applications as drug carriers are still awaiting approval due to insufficient research on their mutagenicity and genotoxicity. Simple and mild preparation of CS-based nanoparticles is always encouraged for ideal drug delivery systems due to their water solubility. In addition, researchers have observed that treating seeds or foliar sprays by applying CS derivatives or nanomaterials demonstrated effective results in enhancing plant growth, increasing crop yields, and advancing food security. This approach not only reduces the need for pesticides and fertilizers but also contributes to environmental pollution reduction and promotes the sustainability of entire agricultural systems. Nevertheless, the detailed molecular mechanisms by which they influence seeds and entire plants remain unclear, underscoring the need for further research into their genomic, proteomic, and metabolic effects.

Looking ahead, the development of novel CS derivatives through the incorporation of biologically active heterocyclic groups using recent techniques and methodologies involving different reaction mechanisms like CO, CN, or CH activation reactions is a highly promising prospect. However, research into the structure–activity relationship is an important factor in developing bioactive derivatives, along with a high degree of substitution of the functional groups. Researchers are consistently encouraged to explore novel reactions in conjugation with the structure–activity relationship. The exact mechanism of modified CS derivatives against antimicrobial activities has not been thoroughly explored; nevertheless, these effects are often generalized with unmodified CS. Advanced research regarding the mechanism of antimicrobial activities due to the specific functional group of CS derivatives is needed. Continuous research and development efforts are needed to enhance the water solubility, mitigate toxicity, and bolster the immune responses and functioning mechanisms of CS-based derivatives for applications in human drug delivery and agrochemicals. Moreover, contemporary technologies like computational and artificial intelligence can be used to serve not only in the design and synthesis of innovative CS derivatives and nanoparticles, but also as a means to approach the most realistic mechanisms for antimicrobial effects, drug delivery systems, and plant growth enhancement.

## Figures and Tables

**Table 1:** Patents on chitosan-based materials in antimicrobial, drug delivery, and agricultural applications.

Patent Area	Application	Final Form	References
Biomedical/Antimicrobial	As a teat sealant wherein CS hydrogels accelerate involution and prevent infection of mammary gland	Hydrogels	[33]
Biomedical/Antimicrobial	An adsorbent biodegradable wound dressing	Films	[34]
Biomedical/Antimicrobial	CS coating in metallic nanoparticles as an anti-biofilm agent on medically implantable articles	Nanoparticles/powder	[35]
Biomedical/Antimicrobial	For coating *Listeria iuanuii*, to enhance negative surface charge and thus immune response	CS oligosaccharide	[36]
Biomedical/Antimicrobial	Antimicrobial wound dressing material with quaternized CS	Quaternized CS sponges	[37]
Biomedical/Antimicrobial	Formulation of CS with metallic nanoparticles for treating skin lesions/infections	Gel matrix	[38]
Biomedical/Drug delivery	CS hydrogel used for targeted drug delivery	Hydrogels	[39]
Biomedical/Drug delivery	Low-toxicity and high-affinity CS derivative for drug delivery	Quaternized, acylated CS derivative	[40]
Biomedical/Drug delivery	Delivery of IL17RC protein that function by improving nasal adsorption	Nanoparticles	[41]
Biomedical/Drug delivery	CS–nucleic acid polyplexes for delivery of nucleic acid encoding IL-2	Nanoparticles	[42]
Biomedical/Drug delivery	Treatment of transected peripheral nerve injuries	CS hydrogel	[43]
Agricultural	Gel microspheres of CS with manganese and prothioconazole for use against pests	Gel microspheres	[44]
Agricultural	Antifungal activity in plants	CS derivative	[45]
Agricultural	Pesticides that comprise CS and berberine	Powder, suspension	[46]
Agricultural	Liquid fertilizer with CS oligosaccharides and amino acids	Liquid	[47]

**Table 3:** Applications of CS-based nano/micro particles as drug/vaccine carriers for infectious pathogens.

CS Derivative/Nanomaterials	Preparation	Interference	References
Hesperidin-loaded CS nanoparticle	Emulsification and evaporation methods	Nasal delivery of the anti-inflammatory HPD compound to inflammatory lungs; Enhance cellular uptake in the inflammatory microenvironment compared with free HPD; Suppress the cytokine storm syndrome and acute lung injury/acute respiratory distress syndrome in a murine model of inflammatory lung disease.	[205]
CS biguanidine nanoparticle	One pot, green, ionic gelation method	Inhibitory activity against the growth of three different *Mycobacterium tuberculosis* pathogens (sensitive, MDR, and XDR) compared with isoniazid drug (standard anti-tuberculosis drug).	[206]
Rifampicin-loaded octanoyl CS nanoparticle	Double emulsion solvent evaporation technique	Pulmonary delivery; Optimization of drug release, solubility, and pulmonary release of rifampicin through this carrier; Aim at improving the interventions for tuberculosis.	[207]
Carvacrol-loaded bovine serum albumin (BSA) CS nanoparticle	Ionic gelation method	Mucosal delivery; Ensure delayed release and limited degradation in the gastric conditions; Effective eradication of *Salmonella enterica* serovar *Typhi.*	[208]
Doripenem-loaded CS micro-particles	Ionic gelation, spray-drying method	Effective pulmonary delivery of doripenem; Significant effect on reducing the infection by *P. aeruginosa.*	[209]
Chlorhexidine-infused CS	One-pot method	Epidermal delivery; Show improved antibacterial activity against *S. aureus* and *S. epidermidis.*	[210]
Ciprofloxacin-loaded CS/poly (2-ethyl-2-oxazoline)	Solvent evaporation method	Antimicrobial properties against *S. aureus* and *E. coli*; Mucoadhesive vaginal drug delivery system for sheep.	[211]
CS nanoparticle loaded with dolutegravir	Spray-drying method	High drug loading and releasing activity; Anti-HIV activity.	[212]
CS nanoparticle loaded with phage endolysin Cpl-1	Ionic gelation method	Mucoadhesive drug delivery; Increased therapeutic applicability of endolysins.	[213]
Magnetic CS loaded with amphotericin B	Chemical coprecipitation method followed by surface coating	Stable and controlled delivery of amphotericin B; Interfere with biofilm formation and fungicidal effect.	[214]
CS-wrapped carbon nano tubes	Cross-linking method of CS and CNTs	Separation, identification, and eradication of rotavirus and *Shigella*, waterborne pathogens in water samples of diverse sources.	[215]
CS/lecithin nanoparticles loaded with antibiotics	Central rotatable composite design method	Epidermal delivery; Increase the accumulation of antibiotics in the epidermis with higher retention ratio compared to the commercial formulations; Test against *E. coli* and *S. aureus.*	[216]
CS–hyaluronic acid-based nanoparticle containing ceftazidime	Ionic gelation method	Mucoadhesive drug delivery; Increase interaction with ocular surface and drug residence time; Prolong drug release profile.	[217]
CS nanoparticle containing LL-37	Ionotropic gelation method	Increase half-life and prolonged LL37 biological activity; Inhibition of high biofilm formation by Methicillin-resistant *S. aureus* when compared to LL37 alone.	[218]
Inactivated PR8 influenza virus-loaded CS alginate and trimethyl CS nanoparticles	Ionic gelation method	Immunoadjuvant potential after nasal immunization; Exhibited higher IgG2a/IgG1 ratio as criteria for Th1-type immune response; Efficient intranasal antigen delivery system for nasal vaccines.	[219]
Inactivated Dengue virus (DENV)-loaded trimethyl CS nanoparticle	Ionotropic gelation method	Highly immunogenic, inducing greater levels of antibodies (total IgG, IgG1, IgG2a and neutralizing antibodies) and T cells (activated CD4⁺ and CD8⁺ T cells) against DENV-2 compared to soluble DENV-2 immunogens.	[220]
CS-based nanoparticle vaccine loaded with *Salmonella Enteritidis* outer membrane proteins and flagellin proteins	Ionic gelation method	Mucosal delivery; Induce specific immune responses against the bacteria and reduced cecal colonization in birds; Almost 65.9% birds which were vaccinated had cleared the infection.	[221]
*E. coli* Nissle 1917 microencapsulated CS–alginate	Ionic gelation method	Reduce *Campylobacter jejuni* colonization in infected chickens; Improve the intestinal morphology, increase immune responses of treated chickens via likely activation of the Th1, Th2, and Th17 pathways.	[222]
Mast cell activator C48/80 with CS nanoparticle	Mixing of C48/80 compound with CS, lyophilization	Exhibit adsorption of model antigen C48/80 and biocompatible onto the nanoparticle; Demonstrate first time for the hepatitis B surface antigen loaded Chi-C48/80 nanoparticle as nasal vaccination strategy.	[223]
Newcastle disease virus (NDV/La Sota) and infectious bronchitis virus (IBV/H120)-loaded quaternized CS (-2-hydroxypropyl trimethyl ammonium chloride CS (*N*-2-HACC) and *N*, *O*-carboxymethyl CS) nanoparticles	Polyelectrolyte composite method	Newcastle disease and Infectious Bronchitis elicit immune response in chickens after intranasal administration; Inducement of higher titers of IgG and IgA antibodies; Increase the proliferation of lymphocytes and induce higher levels of cytokines, including IL-2, IL-4, and IFN-γ.	[224]
CS nanoparticles encapsulating influenza H9N2 HA2 and M2e mRNA molecules	Anionic gelation method	Delivery of immunogenic antigens to antigen presenting cells; Effective penetration of the mucosal barrier to reach immune initiation sites; Efficient release of mRNA molecules into antigen presenting cells; Higher systemic IgG, mucosal IgA antibody response.	[225]

Note: HPD: hesperidin, MDR: multidrug-resistant, XDR: extensively drug-resistant, *P. aeruginosa*: *Pseudomonas aeruginosa*, *S. aureus*: *Staphylococcus aureus*, *S. epidermidis*: *Staphylococcus epidermidis*, *E. coli*: *Escherichia coli*.

**Table 4:** Application of chitosan and its functionalized nanoparticles for drug delivery system.

Form	Drug Carrier System	Drug or Enzymes	Preparation	Interference	References
Nanoparticle	Anti-hMAM and anti-HER2 PEGylated CS nanoparticle	Doxorubicin (DOX)	Mixing the DOX in the PEG-CS solution, cross-linking with TPP.	Cytotoxic against MCF-7 cancer cells (breast cancer).	[226]
	Hyaluronic acid-coated thiolated CS nano particle	Vincristine	Ionic gelation process	In vitro anticancer activity against cancerous prostate cells.	[227]
	Human serum albumin (HSA)-coated trimethyl CS	IR780 (I) or bufalin (B)	Ionic gelation process and electrostatic absorption	Inhibition of cell proliferation and mitochondrion activity of metastatic 4T1 breast cancer cells; Enhance the tumor-targeting and penetrating capability for photo thermal therapy, efficient prevention of lung metastasis of breast cancer.	[228]
	*O*-carboxymethyl CS nanoparticle	Glipizide	Ionotropic gelation method	Significant impact in insulin-resistant/type 2 diabetes.	[229]
	CS nanoparticle	Sitagliptin	Ionic gelation method	Increase symptomatic relief of Alzheimer’s disease by increasing the level of sitagliptin in the brain.	[230]
Film	CS-based film	Brimonidine tartrate (BT)	Dissolution method	High corneal permeability with fast drug release for potential ocular drug delivery.	[231]
Patches	CS, thiolated CS patches with flaxseed oil and coriander oil	Tizanidine	The solvent casting method	In vitro transdermal drug release and drug permeation, skin irritation; In vivo application, pharmacokinetics analysis, and stability studies.	[232]
Hydrogel	Carboxymethyl CS–silk fibroin peptide/oxidized pullulan (CMCS-SFP/OPL) hydrogel-based microneedle	Salvia miltiorrhiza	Polydimethylsiloxane (PDMS) mold	Direct drug release from HFM-1; Alternative transdermal delivery of water-soluble Salvia miltiorrhiza.	[233]
Micelles	CS thiourea with gold nanorods	Paclitaxel	Ring opening polymerization, dialysis, gold-thiolate complex formation	Light-sensitive drug delivery vehicle; Cytotoxicity against MCF7 cells.	[234]
	*O*-CMCTS-VES micelles nanoparticles	Doxorubicin	Dehydrative condensation of the carboxyl group of the carboxymethyl CS and the amino group of the vitamin E succinate, self-assembled nano-micelles	Efficient cellular uptake by HePG2 cancer cells (human liver cancer) and inhibition of tumor (62.7%).	[235]
Liposome	Trimethylated CS-coated flexible liposomes	Resveratrol	Solvent injection method, electrostatic adsorption	Topical drug (Resveratrol) delivery to reduce blue-light-induced retinal damage; Modulation for the mitochondrial membrane potential and protection of ARPE-19 cells from damage by H_2_O_2_.	[236]
	Aptamer-functionalized liposomal coated with calcium alginate/CS/PEC	5- Fluorouracil (AFL5-FU)	Optimized thin film method, freeze–thaw process	Oral formulation for colon cancer therapy; Cytotoxicity against on the colon cancer cell line, HT-29 (IC_50_; 66.53 3M).	[237]
	CS oligosaccharide (CO) nanoparticle based on liposomes	Photosensitizer HPPH and the hypoxia-activated prodrug TH302 (CO-HPPH- TH302/Lipo)	O/W emulsification method, mixing the lipids, drugs, and CO-OA	Activation for PDT and chemotherapy by HPPH and TH302; Inhibition of the growth of CD44-overexpressing tumors with minimal damage to normal tissues by CD44+-targeted liposomes.	[238]

Note: Anti-hMAM: anti-human mammaglobin, Anti-HER2: anti-human epidermal growth factor, HFM-1: Helicase for Meiosis 1, O-CMCTS-VES: carboxymethyl CS/vitamin E succinate nano-micellar System, PEC: photoelectrolyte complexes, HPPH: 2-[1-hexyloxyethyl]-2-devinyl pyropheophorbide-a (Photochlor), TH302: Evofosfamide, CO: CS oligosaccharide, OA: oleic acid, PDT: photodynamic therapy.

## Data Availability

Not applicable.
